# Losing a parent during childhood: The impact on adult romantic relationships

**DOI:** 10.1111/famp.13060

**Published:** 2024-09-19

**Authors:** Carline J. M. van Heijningen, Sheila R. van Berkel, Iris Langereis, Bernet M. Elzinga, Lenneke R. A. Alink

**Affiliations:** ^1^ Institute of Education and Child Studies, Leiden University Leiden The Netherlands; ^2^ Institute of Psychology, Clinical Psychology Unit, Leiden University Leiden The Netherlands; ^3^ Leiden Institute for Brain and Cognition (LIBC), Leiden University Leiden The Netherlands

**Keywords:** attachment style, childhood parental death, parental bonding, parental loss, relationship satisfaction, romantic relationships

## Abstract

The disruption of the parent–child attachment bond due to parental death (PD) may lead to lingering feelings of unsafety or insecurity that might potentially transfer to adult intimate relationships. The aim of the current study was to investigate whether experiencing childhood parental death (CPD) was associated with adult romantic relationship formation and stability, attachment style, and relationship satisfaction, and whether this is dependent on (in)secure parental bonding. In this cross‐sectional study, relationship indicators were assessed using self‐report questionnaires in adults (25–45 years old) who experienced PD during childhood (*n* = 236), in adulthood (*n* = 301), and who did not experience PD (*n* = 278). Experiencing CPD was not associated with relationship formation, relationship stability indicators, and relationship satisfaction. However, individuals who experienced CPD reported higher levels of attachment anxiety and avoidance within their current romantic relationship compared to individuals who did not experience (childhood) PD. Furthermore, insecure bonding with the deceased parent was associated with higher levels of attachment anxiety, while this was not the case for the quality of bonding with the surviving parent or new partner of the surviving parent. These findings on the association between CPD and adult attachment insecurity provide new insight in how attachment insecurity to the deceased parent may be related to attachment insecurity in adult relationships, which is important to discuss when working with individuals who experienced CPD.

The permanent disruption of the attachment bond between parent and child early in life as a result of the death of a parent increases the risk of mental and physical health problems on the short and longer term (e.g., Lytje & Dyregrov, 2019). Given the importance of early attachment relationships for social–emotional development and expectations concerning relationships later in life, the disruption of the attachment relationship between parent and child may also lead to difficulties in close relationships with others later in life (Bowlby, 1980; Luecken & Roubinov, 2012). The death of a parent is an unpredictable and uncontrollable event (Luecken & Roubinov, 2012), which may lead to feelings of unsafety, insecurity, or helplessness experienced by the child (e.g., Biank & Werner‐Lin, 2011; Cavanagh et al., 2008; Fraley & Bonanno, 2004; Schoenfelder et al., 2011; Worden & Silverman, 1996). These feelings of insecurity might also be experienced in intimate relationships, potentially driven by (implicit) expectations and (anxious) feelings of losing another important person in one's life (e.g., Biank & Werner‐Lin, 2011; Lytje & Dyregrov, 2019; Mireault & Bond, 1992; Schoenfelder et al., 2011). This may manifest in avoiding to initiate and/or maintain close or meaningful relationships, anxious feelings and worrying about a potential loss or rejection, and longing for reassurance (described as attachment avoidance and anxiety, respectively; Hazan & Shaver, 1987; Fraley & Shaver, 2000; Walsh, 2003).

Attachment avoidance and anxiety characterize an insecure adult attachment style (e.g., Fraley & Shaver, 2000; Hazan & Shaver, 1987) and are both related to lower levels of relationship satisfaction (e.g., Candel & Turliuc, 2019). Given that insecure adult attachment and low levels of relationship satisfaction are associated with low mental wellbeing and less optimal health outcomes (e.g., Gómez‐López et al., 2019; Proulx et al., 2007; Robles et al., 2014), the experience of childhood parental death (CPD) may also extend to these domains. This underlines the need to investigate relational functioning in adulthood for those who experienced CPD to prevent potential less optimal (mental) health outcomes. Furthermore, more insight into the association between experiencing CPD and relationship outcomes and potential risk or protective factors may also be of clinical relevance to inform important persons as well as healthcare professionals on how to support individuals who lost a parent during childhood. Despite clear theoretical links, empirical evidence on long‐term social and relational functioning after experiencing CPD is scarce. Therefore, the current study aims to investigate the association between experiencing CPD and romantic relationship formation, indicators of romantic relationship stability, adult attachment styles (i.e., anxiety and avoidance), and relationship satisfaction and to shed light on the role of recollections of parental bonding during childhood. The study was informed by a broad systemic perspective, considering multiple relational influences in adaptation after parental loss during childhood (Walsh, 2003, 2023; Walsh & McGoldrick, 2013).

## CPD and romantic relationship outcomes

When focusing on the formation of adult romantic relationships, there is relatively little and only inconsistent empirical evidence for the association between experiencing CPD and relationship formation. Some studies showed an association between CPD and a higher likelihood of getting married and/or cohabitating (at a young age) for women (Gimenez et al., 2013; Høeg et al., 2018; Tebeka et al., 2016), or a higher likelihood of early marriage only when the surviving parent remarried (Teachman, 2004). Other studies found a lower likelihood of getting married before 25–33 years for women who lost their parent during childhood specifically (Feigelman et al., 2017) or an increased likelihood of separation for bereaved men and women (i.e., divorce or no longer living together; Høeg et al., 2018), whereas other studies did not find any associations (Booth et al., 1984; Maier & Lachman, 2000; Teachman, 2004).

It is suggested that the impact of CPD on relationship formation could be due to insecure attachment as a result of CPD (Høeg et al., 2018). Qualitative studies and work based on clinical and anecdotical experiences have described how individuals who experienced CPD may experience anxious feelings within romantic relationships (Edelman, 1994; Koblenz, 2016; Leichtentritt et al., 2018). Additionally, a study described how separation of their romantic partner reminds them of the loss of their parent and how such an event could intensify grief over their parent's death (Meyer‐Lee et al., 2020). In terms of quantitative studies, only a few studies examined the association between CPD and adult attachment styles and relationship satisfaction. These studies found small associations between CPD and an insecure, dismissing attachment style in psychology students (Brennan & Shaver, 1998) or higher levels of attachment anxiety and avoidance within their romantic relationship for mothers who experienced maternal death (Mireault et al., 2002), whereas other studies found no differences with regard to attachment or relationship satisfaction (Brent et al., 2012; Varol et al., 2021). Thus, while there are clear descriptions of insecure attachment after experiencing CPD, it is not clear how common these experiences are among adults with a history of CPD.

## The role of parental bonding

A potential protective factor in the association between the experience of CPD and romantic relationship outcomes later in life is (the perception of) the bond between a parental attachment figure and the child (Bowlby, 1980; Fraley & Shaver, 2000; Hazan & Shaver, 1987; Høeg et al., 2018). The surviving parent is suggested to play a crucial role in how a child is able to cope with and adapt after the death of a parent (Biank & Werner‐Lin, 2011; Høeg et al., 2018; Luecken & Roubinov, 2012; Silverman & Worden, 1992). A recent systematic scoping review showed that positive parenting, open communication, and a more optimal relationship with the surviving parent are associated with more optimal child outcomes, such as lower levels of internalizing and externalizing mental health problems and higher levels of self‐esteem (Jiao et al., 2021). However, to what extent the relationship between the child and the surviving parent after the loss affects romantic relationship outcomes has not yet been examined, either due to the research set‐up (i.e., register‐based study; Høeg et al., 2018) or limited sample size as described by the authors (Mireault et al., 2002). Additionally, the relevance of the child's relationship with the deceased parent for relational functioning has not been studied extensively. The memories and perception of the bonding with the deceased parent might play a role in how someone experiences romantic relationships later in life. To our knowledge, previous literature has not yet examined the association between long‐term romantic relationship outcomes and the relationship with the deceased parent.

Besides the deceased and surviving parent, a potential new romantic partner of the surviving parent may also play a role in the lives of children after parental death (Tennant et al., 1980). When a new partner plays a significant role in the family, they may become a new, additional attachment figure for the child. This might also change interactions and the relationship with the surviving parent, either in positive and in negative ways (e.g., Boerner & Silverman, 2001; Walsh, 2003). Studies with a qualitative or case study design have illustrated that early remarriage can be experienced as negative by a child (Riches & Dawson, 2000) and indicated that the specific role of the new partner, their integration in the family, and conversations about and maintaining a link with the deceased parent are all important for the child's adaptation (Boerner & Silverman, 2001; Riches & Dawson, 2000). Additionally, the relationship between a new partner and the surviving parent provides an opportunity for the child to observe the parent's interpersonal relationship style, and this modeling could be related to the child's relationships later in life (e.g., Bandura, 1977; Rhoades et al., 2012). However, to our knowledge, the child's (in)secure bonding to a new partner of the surviving parent after CPD has not yet been studied with regard to relational outcomes later in life.

## The current study

The first aim of the current cross‐sectional study was to investigate whether experiencing CPD is associated with romantic relationship formation, indicators of relationship stability, and other romantic relationship indicators in adults between the age of 25 and 45 years old who either experienced parental death during childhood (*n* = 236) or adulthood (*n* = 301), or who did not experience parental death (*n* = 278). A comparison group of adults who experienced parental death during adulthood was included to elucidate whether the long‐term associations are specific to parental death in childhood. We explored whether there was an association between experiencing CPD and romantic relationship formation (e.g., whether participants have formed a romantic relationship) and relationship stability indicators (e.g., the total number of romantic relationships after the age of 17 years old and the duration of their (longest) relationship). Furthermore, we hypothesized that individuals who experienced CPD will report higher levels of attachment‐related anxiety and avoidance as well as lower relationship satisfaction than individuals who did not experience CPD. Given the potential impact of parental divorce on adult romantic relationship outcomes (e.g., Fergusson et al., 2014; Mustonen et al., 2011; Roper et al., 2020), we included parental divorce in the analyses as a potential confounding variable. A second aim was to investigate the role of recollections of parental bonding during childhood with the deceased parent, the surviving parent, and a potential new partner of the surviving parent for individuals who experienced CPD in terms of attachment style and satisfaction within their current intimate relationship. We expected that a positive recollection of any of these bonds could be a protective factor, irrespective of the participant's bonding with the other parent(s).

## METHOD

### Participants

Dutch‐speaking adults between the age of 25 and 45 years old were invited to participate via (paid) advertisements on social media. Two different flyers were used to target participants who either had, or had not, experienced parental death. All participants were requested to invite their siblings to participate, since one of the aims of the broader research project was to investigate similarities and differences in both experiences and long‐term outcomes within families. The final sample consisted of 815 individuals across three groups: individuals who experienced CPD and two comparison groups with individuals who did not experience CPD. Participants in the CPD group (*n* = 236) had experienced the death of one of their biological parents between the age of 4 and 17 years old and their other biological parent was still alive. A minimum age of 4 years old was chosen, as experiences before that age are generally more difficult to accurately remember (Tustin & Hayne, 2010). Participants in the adult parental death (APD) group (*n* = 301) had experienced the death of one of their biological parents after the age of 17 and their other biological parent was still alive. The second comparison group consisted of individuals who did not experience PD (no PD group; *n* = 278). Participants were excluded if they had experienced recent parental death (i.e., two years prior to the start of the study), if the deceased parent did not (partly) live in the same household as the participant during childhood, or if the participant lived in a single‐parent family before the age of 4 years old. Based on these criteria, 60 individuals were excluded after screening. An additional 166 participants were excluded based on incomplete data on variables of interest (i.e., parental bonding and/or romantic relationship outcomes), resulting in the final sample of *N* = 815 with complete data. Participants had a mean age of 34.60 years (SD_age_ = 5.86). The majority (86.9%) identified as female, 12.9% as male, and 0.2% as neither female nor male. Most participants identified with the Dutch majority group (98.2%), of whom 3.9% also identified with one or more other ethnic groups. The sample consisted of unrelated individuals (*n* = 707; 86.7%) and 108 individuals (13.3%) who were related as siblings with one or two other participants (52 families; see Supplemental Materials for further details).

### Design and procedure

The current study had a cross‐sectional, retrospective design and was approved by the Ethics Committee of the Institute of Education and Child Studies of Leiden University (registration number ECPW‐2020/272). After registration, participants received an information letter and an online Qualtrics survey link (Qualtrics, Provo, UT). Informed consent was obtained prior to screening. If participants were eligible to participate after screening, they were asked to fill out several questionnaires, which took approximately 20–40 min to complete. Participants were reminded 7 and 14 days after registration if they had not completed the questionnaires. Upon completion, several participants were randomly selected to receive a gift card of 50 euros. In light of the (potential) emotionally sensitive nature of the study, after completing the online questionnaires participants were shown a list of organizations they could contact for (grief‐related) questions or professional care.

### Materials

#### Background information

Demographic information, information regarding their family circumstances during childhood (e.g., parental educational level and job) and regarding (childhood) parental death (e.g., cause of death, age of deceased parent, and changes and support participants experienced in the first year after their parent's death) was obtained.

#### Romantic relationship formation and stability indicators

Romantic relationship formation and relationship stability were assessed using a self‐report questionnaire. Relationship formation was defined as whether someone is currently in a romantic relationship. Relationship stability indicators were the total number of romantic relationships after the age of 18 years old (including the current relationship) and the duration of the current and longest relationship (Schindler et al., 2010; Soons et al., 2009).

#### Adult attachment

To measure attachment style, the self‐report questionnaire Experiences in Close Relationships—Relationship Structures (ECR‐RS) was used (Fraley et al., 2006, 2011). The ECR‐RS consists of nine items, scored on a 7‐point Likert scale, ranging from 1 (*strongly disagree*) to 7 (*strongly agree*). A mean score was obtained for attachment‐related avoidance (six items; e.g., It helps to turn to my partner in times of need) and a mean score for attachment‐related anxiety (three items; e.g., I often worry that my partner doesn't really care for me). Higher scores indicated higher levels of attachment‐related avoidance or anxiety. The reliability and validity are considered good (Fraley et al., 2011). The internal consistency of the scales in the current sample was good (ECR‐RS attachment anxiety: *α* = 0.88; attachment avoidance: *α* = 0.82).

In total, 96.8% of the 666 participants who had a current romantic relationship and identified as male or female were in a heterosexual relationship. If participants were not in a current romantic relationship, they were asked to fill in the questionnaire regarding their ex‐partner. Scores on attachment insecurity related to an ex‐partner (*n* = 113) were significantly higher than for a current partner (*n* = 667), both on attachment‐related anxiety, *t*(778) = 11.65, *p* < 0.001, and avoidance, *t*(778) = 12.91, *p* < 0.001. These differences may be explained by the fact that these relationships were broken up for a reason and/or the ex‐partners' potential attachment pattern. Given these differences, only individuals with a current romantic partner were included in the analyses on attachment.

#### Romantic relationship satisfaction

The 16‐item version of the Couple Satisfaction Index (CSI‐16; Funk & Rogge, 2007) was used to measure participants' satisfaction with their current relationship. The items are scored on different scales, depending on the format of the item(s). First, participants were asked to indicate their degree of happiness about their relationship on a 7‐point Likert scale (from 0 (*extremely unhappy*) to 7 (*perfect*)) and how often they think things are going well within the relationship on a 6‐point Likert scale (from 0 (*never*) to 5 (*all the time*)). Eight other statements regarding the relationship were provided on a 6‐point Likert scale, from 0 (*not at all (true)*) to 5 (*almost completely (true)*). On the last six items, participants were asked to rate how they felt about their relationship on a 6‐point Likert scale (e.g., from 5 (*interesting*) to 0 (*boring*)). A total sum score was obtained (ranging from 0 to 81), in which a higher score indicates a higher level of satisfaction with their current romantic relationship. The internal consistency and validity of the CSI‐16 was considered strong (Funk & Rogge, 2007). The internal consistency in the current sample was excellent (*α* = 0.96).

#### Recollections of parental bonding

The 16‐item version of the Parental Bonding Instrument (PBI; Kendler, 1996; Parker et al., 1979) was used to retrospectively assess participants' perceptions regarding the parent–child relationship before the age of 18 years old with the deceased parent, surviving parent, and new partner of the surviving parent (if applicable). In line with the cut‐off age of CPD used in the current study (i.e., before the age of 18 years old), the instruction was adapted from the original instructions (the PBI is originally filled in over the first 16 years). In a recent publication, three subscales were distinguished, namely Care (e.g., Could make you feel better when you were upset), Lack of Autonomy (e.g., Liked you to make your own decisions), and Overprotection (e.g., Tried to control everything you did; Kullberg et al., 2020). All items were scored on a 4‐point Likert scale, ranging from 1 (*a lot*) to 4 (*not at all*). A higher total score is indicative of lower levels of optimal parental bonding. The original 24‐item version of the PBI is considered a valid measure with good internal consistency and long‐term stability (Kendler, 1996; Wilhelm et al., 2005). The internal consistency of the 16‐item version of the PBI is considered good (Kullberg et al., 2020), but no further information regarding validity is available. In the current sample, the internal consistency for individuals who experienced CPD (*n* = 236) was good to excellent (deceased parent: *α* = 0.86; surviving parent prior to the loss: *α* = 0.90; surviving parent after the loss: *α* = 0.90; new partner of surviving parent: *α* = 0.91).

Participants were asked to fill in the questionnaire regarding the relationship with their mother and father separately. Individuals in the CPD group were asked to report on their relationship with the surviving parent twice (i.e., once regarding the relationship prior to and once after the loss of their deceased parent). Parental bonding with the surviving parent did not significantly differ prior to and after the death of the deceased parent (*t*(235) = −1.94, *p* = 0.053). Parental bonding regarding the surviving parent after the death of the deceased parent was used in the main analyses, as we expected this to be more reliable as it covered a more recent period. To examine the stability of the results, we repeated the analyses with parental bonding prior to the death instead of after the death of the deceased parent. We also explored whether parental bonding with the surviving parent differed from parental bonding with the deceased parent for individuals who experienced CPD. Participants reported relatively more optimal bonding with their deceased parent compared to their surviving parent both prior to and after the loss, *t*(235) = −6.37, *p* < 0.001; *t*(235) = −6.81, *p* < 0.001, respectively (see Table 1). Individuals who experienced CPD were also asked whether their surviving parent had a new romantic relationship during their childhood. When this new partner lived in the same household as participants during childhood, they were also asked to fill in the PBI on their relationship with this new partner of the surviving parent.

**TABLE 1:** Descriptive statistics of demographic information, romantic relationship outcomes, and parental bonding.

	Childhood parental death	Adult parental death	No parental death
	(*n* = 236)	(*n* = 301)	(*n* = 278)
	*M* (*SD*) or %	*M* (*SD*) or %	*M* (*SD*) or %
Age (years)	34.10 (6.01)	35.86 (5.38)	33.68 (6.00)
Gender of participant (% female)	84.32%	87.04%	88.84%
Age at time of parental death (years)^a^	12.10 (3.89)	26.86 (5.87)	n.a.
Gender of deceased parent (% father)	62.29%	57.81%	n.a.
Gender of deceased parent (% same‐sex)	40.68%	43.19%	n.a.
Cause of parental death (%)^b^			
Natural causes/illness	86.02%	72.76%	n.a.
Accidents	5.08%	1.33%	n.a.
Suicide	6.36%	4.98%	n.a.
Unknown or other cause^c^	2.54%	1.33%	n.a.
Parental divorce (% yes)	13.98%	25.25%	28.06%
Highest completed educational level (%)			
Lower	1.69%	3.65%	1.44%
Intermediate	35.17%	35.22%	33.81%
High	63.14%	61.13%	64.75%
Employment status (%)			
Parttime	46.19%	53.16%	49.28%
Fulltime	35.59%	29.90%	32.01%
Employed, number of hours unknown	1.69%	1.33%	1.80%
Unemployed	16.53%	15.61%	16.91%
Monthly net income (%)			
Below 3000 euros	42.37%	43.85%	44.60%
Higher than 3000 euros	47.03%	46.18%	47.84%
Don't know or do not want to answer	10.59%	9.97%	7.55%
Romantic relationship formation			
Currently in a relationship (% yes)	79.24%	83.06%	82.73%
Total number of romantic relationships	2.12 (1.46)	2.32 (1.66)	2.45 (1.73)
Longest relationship duration (years)^d^	10.39 (6.41)	11.37 (6.65)	9.94 (6.25)
Attachment‐related anxiety^e^	2.84 (1.75)	2.49 (1.61)	2.27 (1.42)
Attachment‐related avoidance^e^	2.21 (0.91)	2.17 (1.07)	2.02 (0.98)
Relationship satisfaction^e^	62.91 (14.25)	62.08 (14.75)	61.86 (15.32)
Parental bonding			
Mother and father (mean)	30.81 (7.06)	29.80 (6.34)	31.08 (7.38)
Deceased parent	28.65 (7.45)	n.a.	n.a.
Surviving parent—prior to loss	32.66 (9.77)	n.a.	n.a.
Surviving parent—after loss	33.26 (9.96)	n.a.	n.a.
New partner of surviving parent (*n* = 79)	36.57 (10.67)	n.a.	n.a.

^a^ Missing values for four participants within the adult parental death (APD)‐group.

^b^ Due to a small technical error in the questionnaire, the cause of death was missing for 59 participants within the APD‐group (19.60%).

^c^ Other causes of death are for example death due to crime, side‐effects of medication, or incorrect medical assessment or medical errors.

^d^ Subsample of participants who had at least one romantic relationship after the age of 18 years old (*n* = 780).

^e^ Subsample of participants who currently had a current romantic partner (*n* = 667).

### Statistical analyses

The analyses were conducted using RStudio version 1.3.959 (R Core Team, 2020; RStudio Team, 2020). Initial group comparisons were conducted regarding age, gender, educational level, income, parental divorce, and parental bonding. When the groups differed on one or more sociodemographic variables, this/these variable(s) were included in the analyses to control for possible confounding effects. Additionally, parental divorce was included as a potential confounding variable. Regarding our first research aim, the associations between experiencing CPD and romantic relationship formation, stability, and other relationship indicators were examined. As several participants were siblings nested within families, resulting in dependency within the data, multilevel analyses with a random intercept per family were conducted to take this dependency into account. To obtain standardized coefficients for all predictors, we used the following formula for analyses that included binary/categorical predictors (i.e., parental divorce, gender of the deceased, and the grouping variable): *β*_*j*_ = *B*_*j*_ × (SD(*X*_*j*_)/SD(*Y*); see Buisman et al., 2020). For analyses with only continuous variables, predictors and dependent variables were standardized to also obtain standardized coefficients. For all analyses, both unstandardized and standardized coefficients are reported. First, one generalized multilevel analysis was conducted to examine whether experiencing CPD is associated with relationship formation (i.e., having a current romantic partner or not). Next, five multilevel regression analyses were conducted to examine whether CPD was associated with romantic relationships stability indicators (i.e., total number of romantic relationships and relationship duration) and other indicators of romantic relationships (i.e., attachment‐related anxiety and avoidance and relationship satisfaction; only for those who were currently in a romantic relationship). In these analyses, the group of individuals who experienced CPD was used as a reference group. A Bonferroni correction for multiple testing was applied by dividing *α* by 3 (*α* = 0.017; i.e., three indicators regarding romantic relationship formation and stability and three other indicators regarding current romantic relationships). Regarding our second research aim, for individuals who experienced CPD and had a current relationship, multilevel regression analyses were conducted to investigate the association between parental bonding with the surviving parent, the deceased parent, and a potential new partner of the surviving parent and attachment‐related anxiety and avoidance, and relationship satisfaction. Lastly, we explored the association between the child's age at time of loss and the gender of the deceased parent and attachment‐related anxiety and avoidance, and relationship satisfaction in the model with parental bonding.

## RESULTS

### Initial group comparisons

Descriptive statistics are depicted in Table 1. The three groups did not significantly differ on demographic variables, such as gender (*χ* ^2^(4) = 5.88, *p* = 0.209), educational level (*χ* ^2^(4) = 4.02, *p* = 0.403), employment status (*χ* ^2^(6) = 3.03, *p* = 0.805), and income (*χ* ^2^(4) = 1.71, *p* = 0.789). However, there was a significant difference regarding participants' age in years, Welch's *F*(2, 521.37) = 12.21, *p* < 0.001. Bonferroni post hoc tests for multiple comparisons showed that individuals who experienced APD were significantly older than individuals who experienced CPD (*p* = 0.001) and individuals who did not experience PD (*p* < 0.001), whereas individuals who experienced CPD and individuals who did not experience PD did not significantly differ on age (*p* = 1.000). Additionally, the three groups differed on whether or not they experienced parental divorce during childhood, *χ* ^2^(2) = 15.74, *p* < 0.001, with individuals who experienced APD (25.2%) or did not experience PD (28.1%) having a higher percentage of experiencing parental divorce than individuals who experienced CPD (14.0%). Lastly, parental bonding with participants' mother and father (irrespective of who passed away) did not differ across groups, Welch's *F*(2, 519.92) = 2.87, *p* = 0.058. Age and parental divorce were included in the analyses to control for possible confounding effects, given the group differences and significant correlations with romantic relationship outcomes (Table 2).

**TABLE 2:** Correlations between age, parental divorce, romantic relationship outcomes, and parental bonding.

	*n*	1	2	3	4	5	6	7	8	9	10	11
1. Age	815											
2. Gender	815	0.02										
3. Parental divorce	815	−0.10**	0.02									
4. Currently in a relationship	815	0.05	0.07	−0.04								
5. Total number of relationships	815	0.18***	−0.07	0.12***	0.17***							
6. Longest relationship duration^a^	780	0.57***	0.13***	−0.15***	0.28***	−0.25***						
7. Attachment anxiety^b^	667	−0.08*	0.11**	0.12**	n.a.	0.15***	−0.16***					
8. Attachment avoidance^b^	667	0.01	−0.07	0.11**	n.a.	0.12**	−0.07	0.43***				
9. Relationship satisfaction^b^	667	−0.11**	−0.02	−0.07	n.a.	−0.10**	−0.04	−0.42***	−0.65***			
10. Parental bonding^c^—DP	236	0.05	0.06	0.11	−0.03	0.01	0.02	0.20**	0.14	−0.19*		
11. Parental bonding^c^—SP prior to loss	236	0.15*	0.13*	0.14*	−0.07	0.03	0.05	0.16*	0.16*	−0.20**	0.39***	
12. Parental bonding^c^—SP after loss	236	0.13*	0.17*	0.11	−0.11	0.02	0.03	0.11	0.16*	−0.19**	0.31***	0.88***

Abbreviations: DP, deceased parent; SP, surviving parent.

^a^ Subsample of participants who had at least one romantic relationship after the age of 18 years (*n* = 780).

^b^ Subsample of participants who currently had a current romantic partner (*n* = 667).

^c^ Group of individuals who experienced childhood parental death (*n* = 236).

* *p* < 0.05.

** *p* < 0.01.

*** *p* < 0.001.

### Experiencing CPD and romantic relationship formation and stability indicators

A generalized multilevel analysis showed no difference regarding currently being in a romantic relationship between individuals who experienced CPD and those who experienced APD (*b* = 0.24, *β* = 0.30, *p* = 0.288) and those who did not experience PD (*b* = 0.27, *β* = 0.33, *p* = 0.234). This model did not provide a better fit to the data compared to an empty model (i.e. a model without predictors), *χ* ^2^(4) = 4.86, *p* = 0.302. Additionally, multilevel analyses showed no differences regarding both the total number of romantic relationships and the duration of their longest relationship between individuals who experienced CPD and those who experienced APD (*b* = 0.04, *β* = 0.01, *p* = 0.797 and *b* = 0.13, *β* = 0.01, *p* = 0.785, respectively) and those who did not experience PD (*b* = 0.28, *β* = 0.08, *p* = 0.052 and *b* = 0.06, *β* = 0.00, *p* = 0.894, respectively). These models provided a better fit than an empty model, *χ* ^2^(4) = 50.13, *p* < 0.001 and *χ* ^2^(4) = 319.25, *p* < 0.001, respectively, which can be explained by the significant effects of the covariates age and parental divorce. Age and parental divorce were both not associated with being in a romantic relationship (*b* = 0.02, *β* = 0.32, *p* = 0.183 and *b* = −0.24, *β* = −0.26, *p* = 0.266, respectively), whereas both were associated with the total number of romantic relationships (*b* = 0.06, *β* = 0.21, *p* < 0.001 and *b* = 0.53, *β* = 0.14, *p* < 0.001, respectively) and the duration of the longest relationship (*b* = 0.63, *β* = 0.56, *p* < 0.001 and *b* = −1.29, *β* = −0.08, *p* = 0.005, respectively).

### Experiencing CPD and romantic relationship indicators

Three multilevel regression analyses were conducted to examine the association between having experienced CPD and attachment‐related anxiety and avoidance and relationship satisfaction, when controlling for participants' age and parental divorce. Individuals who experienced CPD had higher levels of attachment‐related anxiety within their current romantic relationship than individuals who experienced APD (*b* = −0.39, *β* = −0.12, *p* = 0.012) as well as individuals who did not experience PD (*b* = −0.67, *β* = −0.20, *p* < 0.001). Additionally, individuals who experienced CPD did not differ on attachment‐related avoidance within their current romantic relationship compared to those who experienced APD (*b* = −0.08, *β* = −0.04, *p* = 0.415), but they did differ on attachment‐related avoidance compared to individuals who did not experience PD (*b* = −0.24, *β* = −0.12, *p* = 0.014). Regarding relationship satisfaction, individuals who experienced CPD did not differ from those who experienced APD (*b* = −0.04, *β* = 0.00, *p* = 0.980) and those who did not experience PD (*b* = −0.73, *β* = −0.02, *p* = 0.623). All models provided a better fit compared to the empty model, *χ* ^2^(4) = 31.00, *p* < 0.001; *χ* ^2^(4) = 15.14, *p* = 0.004; *χ* ^2^(4) = 12.28, *p* = 0.015, respectively. Parental divorce was a significant covariate for all relationship indicators (*b* = 0.55, *β* = 0.14, *p* < 0.001; *b* = 0.31, *β* = 0.13, *p* = 0.001; *b* = −2.85, *β* = −0.08, *p* = 0.043, respectively), indicating that individuals with a history of parental divorce had relatively less optimal relationship outcomes, whereas age was only a significant covariate in the association with relationship satisfaction (*b* = −0.30, *β* = −0.12, *p* = 0.003) and not with adult attachment (anxiety: *b* = −0.02, *β* = −0.08, *p* = 0.054; avoidance: *b* = 0.00, *β* = 0.02, *p* = 0.682).

### The role of parental bonding on romantic relationship indicators

In a subgroup of individuals who experienced CPD and had a current romantic relationship (*n* = 187), we examined the role of parental bonding with the surviving parent and the deceased parent on romantic relationship indicators (i.e., attachment‐related anxiety and avoidance and relationship satisfaction within their current relationship). First, we analyzed parental bonding with each parent in separate models. Parental bonding with the deceased parent was significantly associated with attachment‐related anxiety (*b* = 0.05, *β* = 0.20, *p* = 0.005) and relationship satisfaction (*b* = −0.37, *β* = −0.19, *p* = 0.010), but not with attachment‐related avoidance (*b* = 0.02, *β* = 0.13, *p* = 0.081). Parental bonding with the surviving parent after the loss was not significantly associated with attachment‐anxiety (*b* = 0.02, *β* = 0.12, *p* = 0.107) and avoidance (*b* = 0.02, *β* = 0.17, *p* = 0.021), whereas it was significantly associated with relationship satisfaction (*b* = −0.28, *β* = −0.19, *p* = 0.009). When repeating these analyses with bonding with the surviving parent prior to the loss instead of after the loss, the findings were similar, *b* = 0.03, *β* = 0.17, *p* = 0.023; *b* = 0.02, *β* = 0.17, *p* = 0.021; *b* = −0.30, *β* = −0.19, *p* = 0.007, respectively. All models provided a better fit compared to the empty model (*p*s < 0.024), except for the model that included bonding with the surviving parent after the loss on attachment anxiety (*χ* ^2^(1) = 2.57, *p* = 0.109) and the model that included bonding with the deceased parent on attachment avoidance (*χ* ^2^(1) = 3.04, *p* = 0.081).

**FIGURE 1:**
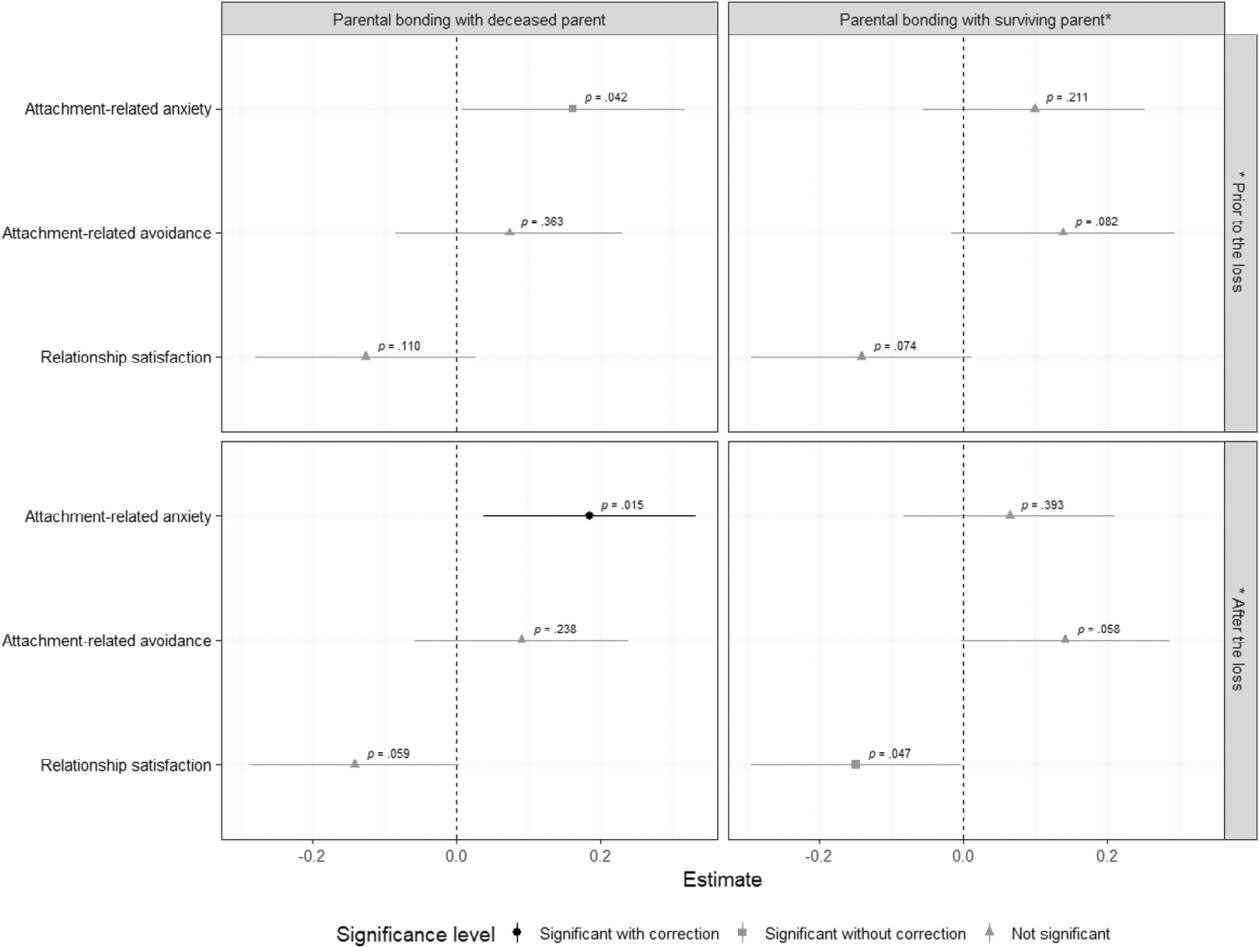
Multilevel analyses of romantic relationship indicators with parental bonding with both the deceased and surviving parent for individuals who experienced childhood parental death (*n* = 187). Within the multilevel regression analyses, all variables were standardized and the estimates refer to beta (*β*). Lower scores of parental bonding are indicative of more optimal parental bonding, higher scores on attachment‐related anxiety and avoidance are indicative of a less secure attachment style, and higher scores on relationship satisfaction are indicative of a higher satisfaction level.

Next, parental bonding with both the surviving parent and the deceased parent was added in the same multilevel model per indicator (see Figure 1). When adding bonding with the deceased parent to the model, parental bonding with the surviving parent after the loss was again not associated with attachment‐related anxiety (*b* = 0.01, *β* = 0.06, *p* = 0.393). Parental bonding with the deceased parent remained associated with lower levels of attachment‐related anxiety (*b* = 0.04, *β* = 0.18, *p* = 0.015) in a model that included parental bonding after the loss. Neither parental bonding with the deceased parent nor the surviving parent after the loss was significantly associated with attachment‐related avoidance and relationship satisfaction (see Figure 1). When repeating these analyses with parental bonding with the surviving parent prior to the loss, findings were similar (see Figure 1), although the association between bonding with the deceased parent and attachment anxiety was no longer significant after correction for multiple testing in a model that included parental bonding prior to the loss (*b* = 0.04, *β* = 0.16, *p* = 0.042). Models on attachment anxiety (*p*s < 0.014) and relationship satisfaction (*p*s < 0.008) provided a better fit compared to the empty model. The model with bonding with the deceased parent and surviving parent after the loss on attachment avoidance provided a better fit compared to the empty model (*χ* ^2^(2) = 6.48, *p* = 0.040), whereas the model with bonding with the deceased parent and surviving parent prior to loss did not (*χ* ^2^(2) = 5.93, *p* = 0.052).

Lastly, we explored the association between the child's age at time of loss and attachment‐related anxiety and avoidance, and relationship satisfaction in the model with parental bonding. Age of the child at time of loss was not associated with attachment anxiety (*b* = −0.03, *β* = −0.06, *p* = 0.419), attachment avoidance (*b* = −0.01, *β* = −0.05, *p* = 0.489), and relationship satisfaction (*b* = 0.14, *β* = 0.04, *p* = 0.596). Furthermore, we explored the association between the role of the gender of the deceased parent and attachment anxiety, attachment avoidance, and relationship satisfaction for female participants who experienced CPD and had a current romantic relationship (*n* = 155) in the model with parental bonding. Gender of the deceased parent was not associated with attachment anxiety (*b* = 0.18, *β* = 0.05, *p* = 0.547) and attachment avoidance (*b* = 0.28, *β* = 0.15, *p* = 0.063), whereas it was associated with relationship satisfaction (*b* = −5.82, *β* = −0.19, *p* = 0.012), indicating that females who lost their mother (vs. father) during childhood were less satisfied with their relationship with their current partner.

### The role of a potential new romantic partner of the surviving parent

In a subgroup of participants who experienced CPD and had a current romantic relationship (*n* = 187), we explored whether the presence of a new partner of the surviving parent during participant's childhood was associated with current relationship indicators. Whether the surviving parent had a new partner (*n* = 98) or not (*n* = 89) was not associated with attachment‐related anxiety and avoidance, and relationship satisfaction, when controlling for parental bonding with the deceased parent and the surviving parent (*b* = −0.08, *β* = −0.02, *p* = 0.765; *b* = −0.24, *β* = −0.13, *p* = 0.066; *b* = −2.15, *β* = −0.08, *p* = 0.289, respectively). Lastly, for those whose surviving parent had a new partner who lived in the same household during their childhood (*n* = 62), parental bonding with the new partner was not associated with attachment‐related anxiety and avoidance, and relationship satisfaction (*b* = 0.02, *β* = 0.08, *p* = 0.371; *b* = −0.01, *β* = −0.08, *p* = 0.491; *b* = 0.06, *β* = 0.04, *p* = 0.719, respectively).

## DISCUSSION

The aim of the current study was to investigate whether experiencing CPD was associated with romantic relationship formation, romantic relationship stability, and attachment style and satisfaction within current romantic relationships in adults between the age of 25 and 45 years old. We found no association between CPD and romantic relationship formation and stability, but did find evidence for associations with the quality of current romantic relationships. Individuals who experienced CPD reported higher levels of attachment‐related anxiety within their current romantic relationship compared to individuals who did not experience CPD (i.e., those who experienced either APD or who did not experience PD) and higher levels of attachment‐related avoidance compared to those who did not experience PD. No differences were found with respect to the satisfaction with their current romantic relationship. A second aim of the study was to investigate the association between recollections of parental bonding during childhood on relationship indicators in individuals who experienced CPD and had a current romantic relationship. We found that more optimal parental bonding was associated with lower levels of attachment‐related anxiety, but not with avoidance and relationship satisfaction. Interestingly, this was mainly driven by the quality of the bond with the deceased parent, even though the effect of the bond with the surviving parent pointed in the same direction. Lastly, parental bonding with a new partner of the surviving parent was not associated with romantic relationship indicators.

### Experiencing CPD and romantic relationship outcomes

In line with previous studies (Booth et al., 1984; Maier & Lachman, 2000; Teachman, 2004) and contrary to others (Feigelman et al., 2017; Gimenez et al., 2013; Høeg et al., 2018; Tebeka et al., 2016), we found no associations between CPD and romantic relationship formation (i.e., whether participants were currently in a romantic relationship) and indicators of romantic relationship stability (i.e., the total number of relationships, and the duration of their longest relationship). The inconsistent findings in the literature may potentially be explained by factors such as the cause of death (Høeg et al., 2018) and differences in age ranges and the operationalization of relationship formation. For example, previous studies operationalized relationship formation as cohabitation and/or (early) marriage, which can be seen as a more explicit form of commitment within a relationship. Additionally, the heterogeneity of our sample due to the broad age range of participants may affect these outcomes, since other age‐ or development‐related factors may also play a role.

A previous study that did find an association with relationship formation suggested that potential insecure attachment styles could play an important role (Høeg et al., 2018), as the attachment bond between the parent and child can be seen as an important blueprint for future relationships with others (e.g., Bowlby, 1980; Fraley & Shaver, 2000; Hazan & Shaver, 1987; Høeg et al., 2018). Our findings show that experiencing CPD was indeed associated with higher levels of attachment‐related anxiety and avoidance, which is in line with previous quantitative and qualitative studies (Brennan & Shaver, 1998; Edelman, 1994; Koblenz, 2016; Leichtentritt et al., 2018; Mireault et al., 2002). The finding that individuals who experienced CPD reported higher levels of attachment‐related anxiety and avoidance within their relationship could potentially be explained by the attachment theory (Bowlby, 1980). Individuals who experienced an early disruption and loss of an important attachment bond with their parent may be more likely to experience (implicit) expectations and (anxious) feelings related to losing an important (attachment) person later in life (e.g., Biank & Werner‐Lin, 2011; Bowlby, 1980). Interestingly, adults who experienced CPD only differed from adults who experienced APD on reported levels of attachment‐related anxiety, whereas they did not differ on attachment‐related avoidance. This may suggest that later attachment‐related anxiety within relationships is specific for experiencing the loss of a parent during childhood, whereas this may not be the case for avoidance.

Although individuals who experienced CPD reported higher levels of attachment insecurity compared to those who did not experience (childhood) PD, we found no differences on relationship satisfaction. This suggests that attachment insecurity is not reflected in lower levels of relationship satisfaction for these individuals. Potentially the disruption of the attachment relationship between parent and child may specially affect adult attachment instead of other relationship indicators such as relationship satisfaction. Additionally, other factors related to the relationship itself contribute importantly to the attachment (in)security and satisfaction, such as the degree to which partners can openly communicate, the emotional support they experience within their romantic relationship, socio‐economic disparities, and a history of divorce. With regard to relationship outcomes, it should be noted that parental divorce was a significant covariate for almost all relationship indicators (except for whether individuals were currently in a romantic relationship), indicating that individuals with a history of parental divorce had relatively less optimal relationship outcomes compared to those who did not experience parental divorce. Thus, future studies on relational functioning should include whether individuals experienced parental divorce along with other important adverse childhood experiences.

### The role of parental bonding

Additionally, we investigated the role of retrospective parental bonding for individuals who experienced CPD. Previous research has mainly investigated the role of the surviving parent after CPD, whereas the relationship with the deceased parent has been studied less extensively. Regarding this relationship with the deceased parent, we found that more optimal retrospective parental bonding was related to lower levels of attachment‐related anxiety, which shows the importance of the role of memories and the perception of the bond with the deceased parent in how someone experiences adult romantic relationships. The finding that more optimal parental bonding is related to less attachment insecurity is in line with previous research concerning parental bonding of individuals who did not experience parental death (e.g., Gittleman et al., 1998; Matsuoka et al., 2006).

Contrary to these findings regarding parental bonding with the deceased parent, the retrospectively perceived quality of the bond with the surviving parent was not significantly associated with adult attachment nor with relationship satisfaction within their current romantic relationship, when controlling for the quality of the bond with the deceased parent. Additionally, we found that whether the surviving parent had a new romantic partner as well as the parental bonding as retrospectively reported by the participant with this new attachment figure was also not associated with these indicators of romantic relationships. No further information was available regarding the nature of the relationship between remarried partners or regarding characteristics and mental health of the new partner of the surviving parent. As a new partner of the surviving parent is not always seen as a parent, future studies could examine other aspects such as the quality of the relationship with this new partner. Lastly, exploratory analyses show that the child's age at the time of loss and the gender of the deceased parent were generally not associated with attachment style and relationship satisfaction within current romantic relationships for female participants, except for the association between the gender of the deceased parent and relationship satisfaction. Given the small subsample of male participants who experienced the loss of either their father (*n* = 20) or mother (*n* = 12) during childhood, we could not test whether the loss of the same‐sex or opposite‐sex parent was associated with relationship outcomes. Future studies could pay attention to gender and gender role associations regarding romantic relationship outcomes.

Other factors might play a more important role in developing optimal or less optimal relationship outcomes, for example the nature of their parent's death (Høeg et al., 2018), previous experiences regarding romantic relationships (Collins, 2003; Madsen & Collins, 2011), support from other important supportive figures (Walsh, 2003), individuals' mental health and potential (individual or couples) therapy, other experiences within the family, such as a history of childhood maltreatment (Colman & Widom, 2004; Shahab et al., 2021; Zamir, 2022), the current relationship with the surviving parent as well as intergenerational trauma. Future research could take these factors into account when examining romantic relationship outcomes. Furthermore, relational functioning could also be investigated more broadly by examining the association between experiencing parental death during childhood and attachment (in)security within other important adult relationships, such as the surviving parent and close friendships (Fraley & Heffernan, 2013). Additionally, as experiencing (implicit) expectations and (anxious) feelings may also extend to losing other important, meaningful relationships next to a romantic relationship (such as close friendships), future research could also investigate the association between experiencing parental death during childhood and social and emotional loneliness (Hazan & Shaver, 1987; Knoke et al., 2010).

## LIMITATIONS AND STRENGTHS

A limitation of the current study is a potential recall bias regarding participants' recollections of parental bonding during childhood (0–18 years) with their deceased parent and surviving parent. Their perception of the bond with the deceased parent may be affected by various factors, such as the age of participants when they lost their parent and the time they had spent with their parent before the loss, but also how others in the child's environment talked about the deceased parent. Interestingly, we found that individuals who experienced CPD reported more positively about the bond with their deceased parent compared to their surviving parent. This is in line with other studies and may indicate idealization of the deceased parent (Canetti et al., 2000; Richter et al., 1992) and/or a potential relative decrease in secure bonding with the surviving parent over time, due to potential difficulties in coping with the death of their spouse or reduced emotional availability. Although some studies found that parental bonding is relatively stable over years (Murphy et al., 2010; Wilhelm et al., 2005) and studies suggest the possibility of a closer bond with the surviving parent after loss (Jiao et al., 2021), parental bonding has—to our knowledge—not yet been investigated longitudinally before and after parental death and into adulthood in individuals who experienced CPD. Thus, although our study shows that parental bonding with the surviving parent does not significantly differ prior to versus after the death when participants retrospectively reported about this bond, it is unclear how the death could change the (perception of the) relationship between children and their surviving parent on a longer term. Future longitudinal studies are needed to further examine this and could consider also including reports by relevant others, such as siblings or the surviving parent, to investigate to what extent potential recall biases may be at stake.

Despite a potential recall bias and various factors that could have affected the perception of parental bonding in the years after the loss, the current perception of participants' bond with their parents is clearly relevant in the context of important attachment relationships later in life. The inclusion of recollections of bonding with different parental figures (i.e., deceased parent, surviving parent, and a potential new partner of the surviving parent) and controlling for potential confounding effects of parental divorce are strengths of the current study. Although the sample is relatively large and included two comparison groups, the generalizability is limited and the findings should be interpreted with caution given the cross‐sectional design, self‐selection/sampling bias, small subgroups regarding the new partner of the surviving parent, and lack of diversity regarding participants' gender (i.e., the large majority of participants identified as female) and ethnic background/identity. Although the Dutch culture may be a representative example of Western‐European culture, it is important to be careful when generalizing the findings to other countries and cultures.

## IMPLICATIONS AND CONCLUSIONS

Taken together, the current study extends findings of previous studies and shows that experiencing the death of a parent during childhood is not associated with romantic relationship formation, indicators of stability, and relationship satisfaction, but that it is related to higher levels of attachment insecurity within current romantic relationships. These findings are also of clinical relevance. Awareness and understanding of the impact of experiencing the death of a parent on attachment (in)security is important to help and inform significant persons in the environment of a child on how to provide a secure base after the death of a parent (Meyer‐Lee et al., 2020). Our finding that a suboptimal bond with the deceased parent is related to more attachment‐related anxiety also underlines that the relationship with the deceased parent should not be ignored. While (adult) children often feel very loyal to a deceased parent, these findings suggest that it is important to (also) discuss potential attachment insecurity to the deceased parent, when working with children or adults who experienced CPD. Therefore, in clinical practice, it may be important to create awareness and acknowledge the relationship with the deceased parent and the way an individual continues to perceive this internal, mental bond with the parent and with their romantic partner, while taking into account children's unique narratives and experiences in their current relationships.

## Supporting information


Appendix S1.

## Data Availability

Our data include highly sensitive data that could identify participants. Therefore, the data are not openly available in a public repository. However, data are available via the principal investigator of the current research project (L. R. A. Alink), upon reasonable request.
