# Supplementary material for: Losing a parent during childhood: The impact on adult romantic relationships
Source: Fam Process. 2024 Sep 19;64(1):e13060. doi: 10.1111/famp.13060 (PMC11786250; doi:10.1111/famp.13060)
Supplement: Supplementary file 1 — Appendix S1. [file FAMP-64-0-s001.docx]

**Supplemental Material**

**Sibling Identification Procedure**

The sibling identification procedure was performed in RStudio version 1.3.959 (RStudio Team, 2020; R Core Team, 2020). Siblings within families were matched based on date of birth and gender, which they reported about themselves and their siblings. If there was a match based on date of birth and gender, we compared two other variables that should be similar for biological siblings within the same family, namely whether or not one of their biological parents passed away and the gender of the deceased parent (for individuals who experienced CPD and APD). This yielded a list of potential sibling matches. Next, the mutuality of the potential sibling match was examined (i.e., the participants both report about each other as siblings and the relationship type matched (i.e., biological siblings, stepsiblings, half siblings, foster siblings, or adopted siblings)). This yielded a list of mutual sibling matches. When there was a match between participants based on the procedure described above, these siblings were assigned a family ID number. An additional variable was assigned to indicate which and how many siblings within a family participated in the study.

After matching the siblings in R, a manual check was performed in two steps. First, all potential sibling matches were manually and independently checked by (undergraduate) researchers. Mismatches were analyzed to check for deviations/potential errors in the provided information, for example incorrect date of birth (e.g., plus/minus one day, one month, and/or one year, or last two numbers of birth year reversed), a mismatch based on a sibling’s gender or relationship type, etc. The second step was an additional manual check, if participants indicated that they participated via one of their siblings or if participants indicated (in a follow-up part of the research project) whether they knew/thought that their sibling(s) participated, three researchers independently checked whether these participants were matched with another participant in the dataset. The same researchers checked whether deviations (e.g., due to a mistake or typing error) in date of birth were observed that caused mismatches between siblings within the same family. For these participants, the age of the participants and their sibling(s) at time of parental death was used as an extra control variable for participants who experienced CPD and APD, which was manually checked. Additionally, for all participants, a set of other control variables relating to the childhood family was used (i.e., parents’ educational level and job status and whether participants’ parents divorced during childhood). The sibling/family mismatches were discussed and agreement was reached by the three independent coders. When no agreement was reached, the first author was asked to make a final decision. In total, 108 participants within 52 families were identified (48 families with 2 participating siblings and 4 with 3 participating siblings). Within the CPD group, 19 families were included (13 deceased fathers (DF); 6 deceased mothers (DM)); 18 families were included within the APD group (10 DF; 8 DM); 6 families included participants who experienced CPD and APD (1 DF; 5 DM); and lastly, 9 families were included within the no PD group.
